# Characterization of the Ryanodine Receptor Gene With a Unique 3′-UTR and Alternative Splice Site From the Oriental Fruit Moth

**DOI:** 10.1093/jisesa/iev148

**Published:** 2016-02-05

**Authors:** L. N. Sun, H. J. Zhang, L. F. Quan, W. T. Yan, Q. Yue, Y. Y. Li, G. S. Qiu

**Affiliations:** Research Institute of Pomology, Chinese Academy of Agricultural Sciences, Xingcheng, Liaoning 125100, People’s Republic of China (slncaas@163.com; zhanghuaijiang@sina.com; 572925397@qq.com; ywtipcaas@163.com; yueqiang@caas.cn; lyy4455@163. com) and

**Keywords:** ryanodine receptor, *Grapholita molesta*, alternative splice site, expression

## Abstract

The ryanodine receptor (RyR), the largest calcium channel protein, has been studied because of its key roles in calcium signaling in cells. Insect RyRs are molecular targets for novel diamide insecticides. The target has been focused widely because of the diamides with high activity against lepidopterous pests and safety for nontarget organisms. To study our understanding of effects of diamides on RyR, we cloned the RyR gene from the oriental fruit moth, *Grapholita molesta*, which is the most serious pest of stone and pome tree fruits throughout the world, to investigate the modulation of diamide insecticides on RyR mRNA expression in *G. molesta* (*Gm*RyR). The full-length cDNAs of *Gm*RyR contain a unique 3′-UTR with 625 bp and an open reading frame of 15,402 bp with a predicted protein consisting of 5,133 amino acids. *Gm*RyR possessed a high level of overall amino acid homology with insect and vertebrate isoforms, with 77–92% and 45–47% identity, respectively. Furthermore, five alternative splice sites were identified in *Gm*RyR. Diagnostic PCR showed that the inclusion frequency of one optional exon (f) differed between developmental stages, a finding only found in *Gm*RyR. The lowest expression level of *Gm*RyR mRNA was in larvae, the highest was in male pupae, and the relative expression level in male pupae was 25.67 times higher than that of in larvae. The expression level of *Gm*RyR in the male pupae was 8.70 times higher than in female pupae, and that in male adults was 5.70 times higher than female adults.

Diamide insecticides, a novel class of insecticides, were developed in the 1990s (Lahm et al. 2005, Teixeira and Andaloro 2012). Currently, the commercialized diamide insecticides include one phthalic diamide (flubendiamide) and two anthranilic diamides (chlorantraniliprole and cyantraniliprole). The former was discovered by Nihon Nohyaku and codeveloped with Bayer; the later two were synthesized by DuPont and cocommercialized by DuPont and Syngenta. These insecticides have notably high activity against Lepidopteran species and are relatively safe to these insects’ natural enemies and mammals (Tohnishi 2005, Gopal and Mishra 2008, Dinter et al. 2009, Ioriatti et al. 2009, Tiwari and Stelinski 2012). The selectivity of diamide insecticides toward insects over mammals was determined to be due to the different isoforms of ryanodine receptors (RyRs), the target of diamides, in insects and mammals (Sattelle et al. 2008).

RyRs, the largest known calcium channel protein, are named after ryanodine, a plant alkaloid. The receptors are homomeric tetramers with a large hydrophilic N-terminal domain and a small membrane-spanning C-terminal domain. Each monomer of ∼550–580 kDa consists of ∼5,000 amino acids. This channel is located on the endoplasmic reticulum of muscles, neurons, and many other cell types (Ogawa and Murayama 1998, Fill and Copella 2002, Hamilton 2005, Sattelle et al. 2008). RyRs mediate many cellular and physiological activities by regulating the release of Ca^2+^ from the lumen of the sarcoplasmic and endoplasmic reticulum to the cytosol of muscle and nonmuscle cells, causing such effects as neurotransmitter release, hormone secretion, gene expression, and muscle contraction (Ogawa and Murayama 1998). Three RyR isoforms have been indentified in mammals. RyR1 is primarily observed in skeletal muscle, RyR2 is expressed with high levels in cardiac muscle, and RyR3 is found in many tissues with low abundance but is relatively abundant in the brain and diaphragm. Two isoforms (RyRα and RyRβ) were found in fish, amphibians, and birds (Ottini et al. 1996, Ogawa et al. 1999). However, only one RyR isoform exists in such insects as *Drosophila melanogaster*, *Plutella xylostella, Sogatella furcifera*, *Leptinotarsa decemlineata*, *Ostrinia furnacalis*, *Cnaphalocrocis medinalis*, *Carposina sasakii,* etc. (Xu et al. 2000; Sun et al. 2012; Wang et al. 2012a, 2013b; Yang et al. 2014; Sun et al. 2015a,b). These insects RyRs have ∼45–47% amino acid sequence identity with the three mammalian RyR isoforms (Sattelle et al. 2008).

Diamides are mainly used to control insects that attack food crops and vegetables (Ioriatti et al. 2009; Han et al. 2012; Roditakis et al. 2013; Campos et al. 2015). *P. xylostella* has shown high resistance to chlorantraniliprole and flubendiamide because of the high frequency of diamide use in southern China and certain countries in Southeast Asia (Troczka et al. 2012; Wang et al. 2013a; Lin et al. 2013; Guo et al. 2014a,b; Yan et al. 2014). However, these insecticides have not been widely used in controlling the pests of tree fruits. Chlorantraniliprole was reported to have shown excellent pest control activity on the oriental fruit moth (Jones et al. 2010), *Grapholita molesta*, a widely distributed pest that attacks stone and pome tree fruits. *G. molesta* was thought to originate from northwestern China, this pest is currently distributed throughout most peach, pear, and apple orchards in China (Zheng et al. 2013). To date, there have been no published reports of diamides with cross-resistance to the current insecticides. However, with the application of these chemicals for *G. molesta*, the problem of resistance may occur in the future. To clear the characterization of the receptors, the full-length RyR cDNA from *G. molesta* (*GmRyR*) was isolated and characterized, and the *GmRyR* mRNA expression pattern was investigated.

## Materials and Methods

A laboratory colony of *G. molesta* was originally collected in 2012 from Xingcheng in Liaoning Province, China. Larvae were reared on immature apples without pesticides and an agar-free semiartificial diet in a laboratory for several generations under a photoperiod of 15:9 (L:D) h at 25 ± 1°C and 70–80% RH.

**Fig. 1.**
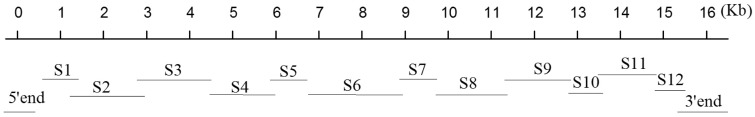
PCR amplification and cloning of *Gm*RyR cDNA.

Total RNA was extracted from larvae, pupa, and adults according to the instructions in the RNAprep pure Tissue Kit (TIANGEN, China). The RNA pellet was dissolved in ddH_2_O. First strand cDNA was synthesized from 1 μl total RNA (650 μg/ml) using the Takara cDNA Synthesis Kit (Takara, China) following the manufacturer’s instructions. Fourteen fragments were amplified to obtain the full-length cDNA of *GmRyR* (Fig. 1).

Degenerate primers were designed by the method of Sun et al (2012, 2015a,b); specific primers were designed based on the obtained sequences (Table 1). The 5′and 3′ ends were amplified with nested PCR, with adaptor primers provided by the SMARTer RACE cDNA Amplification Kit (Clontech, Japan). Amplification of each fragment was performed with the following steps: an initial denaturing step at 94°C for 1 min followed by 30 cycles of 98°C for 10 s, 48–65°C (determined by the Tm of primers) for 30 s, and 72°C for 1–3 min (depending on the length of the amplified fragments), ending with an additional polymerization step at 72°C for 5 min. The PCR products of all fragments were purified and subcloned into the pMD19-T Simple Vector (Takara). JM109 (*E. coli*) competent cells transformed by the recombinant plasmids produced earlier were inoculated. The positive recombinant clones were then sequenced by BGI (Beijing, China).

**Table 1.** Primers used in cloning *G. molesta* RyR cDNA

Name	Primer namen	Primer sequence (5′–3′)	Description (length bp)
S1	F1	TTYCAYGTRACNCAYTGGTC	RT-PCR product S1 (824)
	R1	TGYTTYTCYTCGTGYTCCAT	
S2	F2	TTCCGTGAACCTTGGCGAGA	RT-PCR product S2 (1,431)
	R2	GCAGGGATGTATCTTGTGGA	
S3	F3	TGAGTTGCCGCTTCCTTCTT	RT-PCR product S3 (1,604)
	R3	CATCTGTTGGCGTTCCTTGA	
S4	F4	GTAYACVAARGAYCARCCCAT	RT-PCR product S4 (1,482)
	R4	TCACCATCTCGCCAAGGTTCAC	
S5	F5	MRDCCRCAYCARTGGGCTAG	RT-PCR product S5 (842)
	R5	GCRCCYTCVGCCATYTTCAT	
S6	F6	CGAAAACTTGTTCCTGCCTC	RT-PCR product S6 (2,383)
	R6	ATACTCCGACAGCCGTGACA	
S7	F7	CGHGARGCKGTBTCMGACTT	RT-PCR product S7 (854)
	R7	CKYTCVGCCATRTTYTGCAT	
S8	F8	ACGGATTCAGCGACCCCATT	RT-PCR product S8 (1,394)
	R8	TGGTTGCCTTGAGTGGGAGT	
S9	F9	TCWTAYTTRCCGTTCTGGTG	RT-PCR product S9 (1,586)
	R9	ATGTGGAGCAGCACCATCTC	
S10	F10	ATMCAYGARCAAGARATGGA	RT-PCR product S10 (824)
	R10	CCTTCNARCATNGAHARCATCAT	
S11	F11	AGTTGTCCAAGCACTCCTCG	RT-PCR product S11 (2,142)
	R11	CTCTTCGTGAGCCGCAAATG	
S12	F12	TGGGACAARTTYGYRAAGAA	RT-PCR product S12 (716)
	R12	ATRAARCARTTGGAYTCCATGT	
3′end	3′OF	CTGTGACGCATAATGGGAAGCA	RT-PCR product 3′RACE (1,143)
	3′IF	AAGAGGACGACGAGGTCAACAG	
5′end	5′OR	AGAATCGCAGCACATCCCCACCG	RT-PCR product 5′RACE (977)
	5′IR	GGCTTGCGACATTACTGACCCTCC	
RyR	RyR-F	GCTTCACCCGACGAGGCAGTGGAA	qRT-PCR (97)
	RyR-R	CTTGTGCTTGCTTCTTCGCTTGTTCTC	
GAPDH	GF	GCCAGCTACGACGCCATCAAGCA	qRT-PCR (109)
	GR	CGCCGATGAAGTCAGAGGACACG	
ASP-a	PaF	GAGCGAGCAGGATGATGTTT	diagnostic PCR for the presence of exon a
	PaR	AATTTTCTTTGCCGGTCTCG	
ASA-a	AaF	TCCGAGACCGGTAAAGGCA	diagnostic PCR for the absence of exon a
	AaR	CCGTCGTGATGTGTCGTATG	
AS-b	bF	TACAGCGGTAGTACAGAGTCG	diagnostic PCR for exon b
	bR	TCGTATCTGTGGGTTAGGAC	
AS-c	cF	CACCGCGGGTCGACGGAAAGT	diagnostic PCR for exon c
	cR	TCGTATCTGTGGGTTAGGAC	
ASP-d	PdF	GCCCAGTACAGCAGGTCAAG	diagnostic PCR for the presence of exon d
	PdR	AAGGCGTGGACTTGTAGCGA	
ASA-d	AdF	AGTGTCACAG ACGAACCTCA	diagnostic PCR for the absence of exon d
	AdR	AAGGCGTGGACTTGTAGCGA	
ASP-e	PeF	CAGATGTCGTGACGGATTCA	diagnostic PCR for the presence of exon e
	PeR	GGTGAGGAGGTCGTATGGGA	
ASA-e	AeF	GTCTGGTGGC ACGGATTCAG	diagnostic PCR for the absence of exon e
	AeR	GGTGAGGAGGTCGTATGGGA	
ASP-f	PfF	TACTCGTTCTATCCGCTGCT	diagnostic PCR for the presence of exon f
	PfR	AGCTCCGATTTTATGAGCCG	
ASA-f	AfF	TCTTTACAGCAAACTGGGTT	diagnostic PCR for the absence of exon f
	AfR	CCTCTTGTCCGATGTTCTCT	

Nucleotide sequences of full-length *GmRyR* cDNA were assembled by overlapping the 14 amplified fragments. *Gm*RyR characterization was conducted using the same methods as performed in *P. xylostella, Spodoptera exigua,* and *Ca.* *sasakii* (Sun et al. 2012, 2015a,b).

The relative expression abundances of five *Gm*RyR samples during three developmental stages (larvae, pupae, and adults) were measured using quantitative real-time PCR. Total RNA was extracted from whole bodies of 10 insects of each sample using the same method as earlier. First-strand cDNA was synthesized from 1,000 ng RNA using the High Capacity cDNA Reverse Transcription Kit (ABI, USA). Real-time qPCR for the RyR gene and GAPDH gene as an endogenous control from *G. molesta* were carried out in 20 µl reaction volumes containing 1 µl cDNA (200 ng/µl), 10 µl SYBR Premix Ex Taq (KAPA, USA), 0.4 µl each of forward and reverse primers (10 mM) (Table 1), and 8.2 µl ddH_2_O using the ABI 7500. Real-time PCR System (Applied Biosystems) in the same amplification condition: 95°C for 3 min followed by 40 cycles at 95°C for 3 s and 60°C for 20 s. Three biological replicates per sample were examined. The amplification efficiency of RyR and GAPDH were estimated by using *E* = (10^−1/slope^) − 1, where the slope was derived from the plot of the cycle threshold (Ct) value versus the log of the serially diluted template concentration, and computed to be 0.9584 and 0.9742, respectively. The data analysis model for quantification of the transcript level of *GmRyR* was computed according the 2^−^*^Δ^^Δ^*^Ct^ method (Schmittgen and Livak 2008). The *GmRyR* expression data were shown as means ± SD. A statistical analysis was performed by Duncan’s multiple range test for significance (*P* < 0.05) using SPSS 20 (SPSS, Inc., Chicago, IL).

Diagnostic PCR analyses for the detection of alternative exons were used to detect the presence of each putative alternative exon in individual cDNA clones. Diagnostic PCR was used to determine the usage of each putative alternative exon for *GmRyR* mRNA from mature larvae, 4-d-old pupae (female and male), and 4-d-old adults (female and male). Data were collected from sets of 30 positive clones for each fragment and developmental stage. Table 1 lists the names and nucleotide sequences of primers used in diagnostic PCR reactions.

## Results

The full-length cDNA of *GmRyR* (GenBank KM034750) is 16,299 bp long, as analyzed by overlaying all of the amplified sequenced fragments. The cDNA contains a 272 bp 5′-UTR, a unique 625 bp 3′-UTR with a 29-bp polyA tail, and an open reading frame (ORF) of 15,402 bp. From the ORF, a protein of 5,133 amino acid residues with molecular weight of 580.00 kDa is encoded with a predicted isoelectric point of 5.40.

**Fig. 2.**
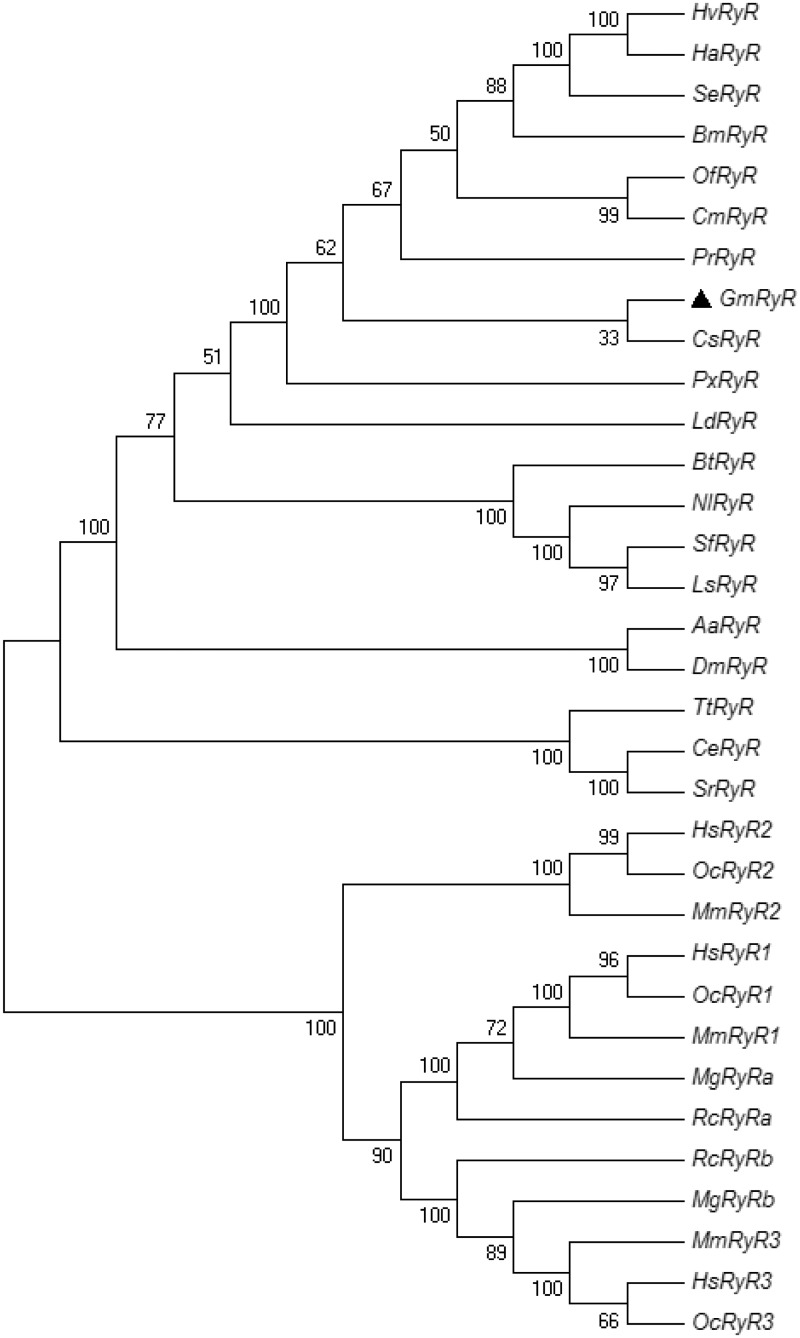
Phylogenetic tree of *Gm*RyR and 32 other RyR isoforms. The *Gm*RyR amino acid sequence was aligned with 32 representative RyR isoforms. The corresponding GenBank accession numbers are as follows: *Heliothis virescens* (*HvRyR*) ADE98118.1; *Helicoverpa armigera* (*HaRyR*) AIA23855.1; *Sp. exigua* (*SeRyR*) AFC36359.1; *B. mori* (*BmRyR*) DJ085056.1; *O. furnacalis* (*OfRyR*) AGH68757.1; *C. medinalis* (*CmRyR*) AFI80904.1; *Pieris rapae* (*PrRyR*) AGI62938.1; *G. molesta* (*GmRyR*) KM034750; *Ca. sasakii* (*CsRyR*) AHN16453.1; *P. xylostella* (*PxRyR*) AEI91094.1; *L. decemlineata* (*LdRyR*) AHW99830.1; *Bemisia tabaci* (*BtRyR*) AFK84957.1; *Nilaparvata lugens* (*NlRyR*) AIA23857.1; *S. furcifera* (*SfRyR*) AIA23859.1; *Laodelphax striatella* (*LsRyR*) AIA23858.1; *Aedes aegypti* (*AaRyR*) XP_001657320.1; *D. melanogaster* (*DmRyR*) AAM71083.1; *Trichuris trichiura* (*TtRyR*) CDW52896.1; *Caenorhabditis elegans* (*CeRyR*) BAA08309.1; *Strongyloides ratti* (*SrRyR*) CEF62113.1; *Homo sapiens* (HsRyR2) NP_001026.2; *Or. cuniculus* (*OcRyR2*) NP_001076226.1; *Mus musculus* (*MmRyR2*) NP_076357.2; *H. sapiens* (*HsRyR1*) NP_000531.2; *Or. cuniculus* (*OcRyR1*) NP_001095188.1; *M. musculus* (*MmRyR1*) NP_033135.2; *Meleagris gallopavo* (*MgRyRa*) ABY50125.1; *Rana catesbeiana* (*RcRyRa*) BAA04646.1; *R. catesbeiana* (RcRyRb) BAA04647.2; *Me. gallopavo* (*MgRyRb*) ABY50126.1; *M. musculus* (MmRyR3) NP_808320.2; *H. sapiens* (*HsRyR3*) CAA04798.1; *Or. cuniculus* (OcRyR3) NP_001076231.1. The neighbor-joining tree was generated in MEGA 6.0 with 1,000 bootstrap replicates.

*Gm*RyR showed amino acid identities with *Cs*RyR, *Se*RyR, *Bm*RyR, *Of*RyR, *Px*RyR, *Sf*RyR, and *Dm*RyR of 92, 93, 91, 93, 91, 79, and 79%, respectively. However, the amino acid identities of *Gm*RyR with *Oryctolagus cuniculus* RyR1–3 was only 44–46%, and the amino acid identity of *Gm*RyR with *Ce*RyR was only 46% as well. To investigate the evolutionary relationships between *Gm*RyR and 32 other RyR isoforms from 25 species, a phylogenetic analysis was performed using ClustalW and MEGA 6.0 based on the ORF amino acid sequences with high bootstrapping support in 1,000 replications (Fig. 2). The phylogenetic tree showed that insect RyRs are well segregated from invertebrate and vertebrate RyRs. *Gm*RyR was clustered with *Cs*RyR from the peach fruit moth, suggesting these two genes are closely related. A number of nucleotide differences were observed between these two overlapping clones. A total of 29 nucleotide differences resulted in 26 amino acid polymorphisms (Table 2). These polymorphisms were located both in the N- and C-terminal regions of *Gm*RyR and may represent different alleles or few errors committed during the PCR procedure.

**Table 2.** Nucleotide and amino acid polymorphisms of *Gm*RyR

Nucleotide position*	Nucleotide exchange	Amino acid exchange^#^
10	G→A	A^4^→R
11	C→G	A^4^→R
158	A→G	G^53^→D
190	G→A	V^64^→M
520	T→C	S^174^→P
614	C→T	L^205^→P
659	T→C	F^220^→G
660	C→G	F^220^→G
662	G→T	G^221^→V
663	T→G	G^221^→V
1,115	G→A	R^372^→K
2,047	A→G	K^683^→E
2,186	A→G	N^729^→S
2,300	G→A	S^767^→N
2,798	C→T	P^933^→L
4,050	C→T	T^1350^→M
5,193	C→T	K^1726^→R
6,262	G→T	E^2154^→D
6,686	G→A	G^2229^→D
6,700	G→T	G^2234^→W
6,965	C→T	T^2322^→M
7,048	C→T	L^2350^→F
8,192	T→C	V^2731^→A
8,705	G→A	C^2902^→Y
12,119	A→G	Y^4040^→C
13,544	A→C	Q^4515^→P
13,545	G→A	L^4516^→S
14,024	T→C	V^4675^→A
15,061	A→G	I^5021^→V

*The number of A in the initial methionine codon represents 1.

^#^The number of amino acid M in the initial ORF represents 1.

Conserved domains of *Gm*RyR were predicted with four types of domains in the N-terminal region. The MIR (Mannosyltransferase, IP3R and RyR) domain was found at positions 217–398. Two RIH (RyR and IP3R Homology) domains (445–654, 2,235–2,462), three SPRY (SPla and RyR) domains (653–805, 1,085–1,217, 1,540–1,692), and four repeated RyR domains (859–953, 972–1,066, 2,836–2,929, 2,962–3,050) were also predicted. Six essential hydrophobic transmembrane domains (TM1–TM6) were predicted in the C-terminal region between amino acids 4,471 and 5,032, specifically at positions 4,471–4,493, 4,463–4,685, 4,743–4,765, 4,885–4,907, 4,933–4,955, and 5,013–5,032. Additionally, a RIH-associated domain (residues 4,009–4,134) and an EF-hand pair (Ca^2+^-binding sites, residues 4,222–4,270) were predicted at the C-terminal region of *Gm*RyR.

**Fig.3.**
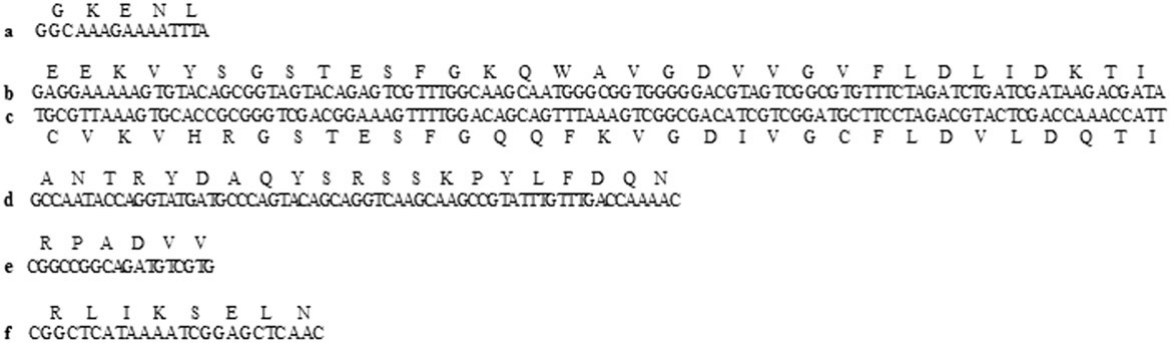
Nucleotide and putative amino acid sequences of alternatively spliced exons in the *Gm*RyR gene.

Five putative alternative splice sites in *GmRyR* were revealed by the alignment of multiple cDNA clone sequences and the sequences of PCR products. They were found between nucleotides 265 and 279 (a), 3,421 and 3,519 (b/c), 4,459 and 4,521 (d), 8,845 and 8,862 (e), and 11,095 and 11,118 (f), respectively. Alternative splicing of nucleotides 3,421 and 3,519 forms one pair of mutually exclusive exons (b/c). Figure 3 shows the nucleotide and inferred amino acid sequences of the six alternative exons identified in this study.

**Fig. 4.**
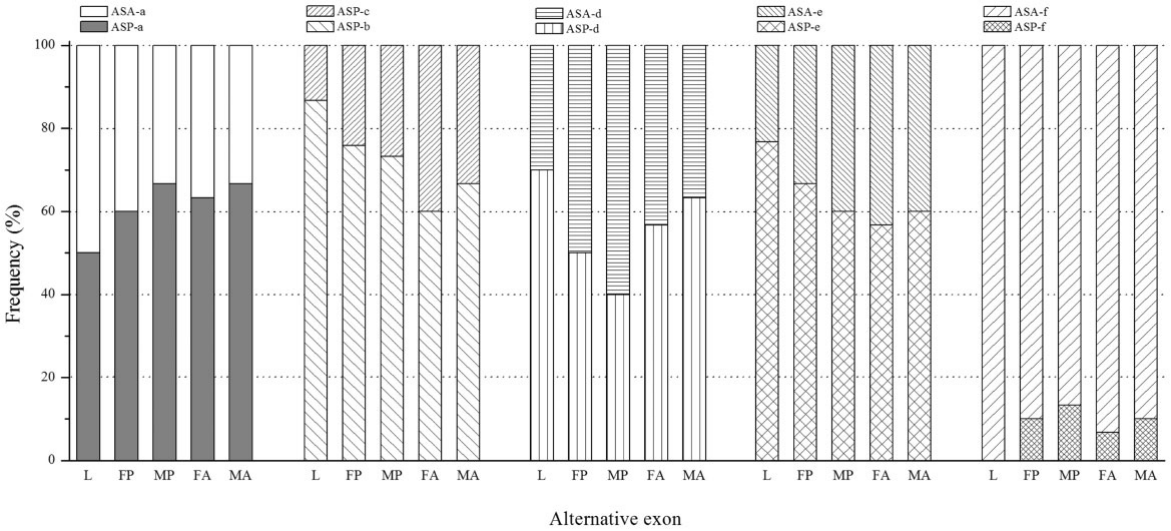
Relative frequencies of individual *Gm*RyR alternatively spliced exon usages. Alternative splicing exons were detected in fourth instar larvae (L), female pupae (FP), male pupae (MP), female adult (FA), and male adult (MA). ASA-a means that alternative splicing exon a is absent, ASP-a means that alternative splicing exon a is present. And other alternative exons (ASP-b, ASP-c, ASA-d, ASP-d, ASA-e, ASP-e, ASA-f, and ASP-f) were also showed in the same method.

Diagnostic PCR analyses for the usage frequencies of each alternative exon in *GmRyR* mRNA are shown in Figure 4. The results show that the usage of exon f exhibited marked developmental regulation. Exon f was not present in all cDNA clones examined from larvae cDNA pools and was only present at low frequencies (6.67–13.33%) in the pupal and adult body cDNA pools.

**Fig. 5.**
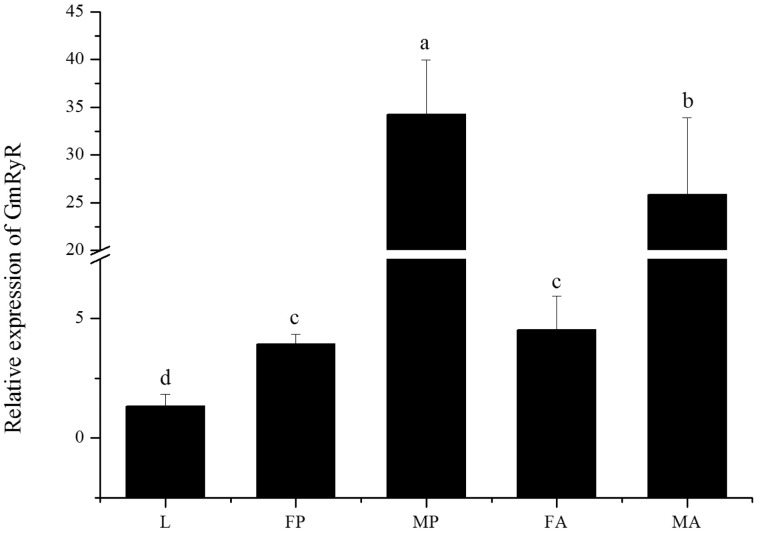
*Gm*RyR relative expression level in fourth instar larvae (L), female pupae (FP), male pupae (MP), female adult (FA), and male adult (MA). The lowercases on the top of each bar means the statistic differences (*P* < 0.05).

The relative expression level of *GmRyR* showed significant variation between different developmental stages and sexes (Fig. 5). The results showed that *GmRyR* had the lowest expression level in larvae and the highest in male pupae, and the relative expression level in male pupae was 25.67 (*P* < 0.001) times higher than that of in larvae. In addition, the expression level of *GmRyR* in male pupae was 8.70 (*P* = 0.001) times higher than in female pupae, and the expression level of *GmRyR* in male adults was 5.70 (*P* = 0.011) times higher when compared with that of female adults.

## Discussion

Ryanodine was isolated from extracts of the stem and root of *Ryania* Speciosa Vahl and exhibited high insecticidal activity to insects and mammals in 1948 (Rogers et al. 1948). A series of experiments found that ryanodine led to muscle contraction by regulating Ca^2+^ release. Thus, RyR was named after this alkaloid (Pessah et al. 1985). However, ryanodine was banned from use in pest control because of its toxicity to mammals. Although insect RyRs have been used as targets of insecticides since ryanodine was isolated, little research about RyRs was reported until the development of diamide insecticides. The diamide insecticides activate the calcium release channel that sensitize to ryanodine in insect and show high selectivity toward Lepidopteran insect pests over mammalian (Cordova et al. 2006). The diamides could evok typical symptoms for insect including poisoning, body contraction, feeding cessation, paralysis, and subsequent mortality. Because diamides are nonreactive in mammals, RyR isoforms from insects have been popular diamide targets, with increasing interest from the pesticide field since the 2000s. Recently, full-length RyRs from insects were cloned, namely *PxRyR, CmRyR, OfRyR, BdRyR SfRyR*, *LdRyR,* and *SeRyR* (Sun et al. 2012, 2015a; Wang et al. 2012a, 2013b, 2014; Yuan et al. 2014; Yang et al. 2014). The unique 625 bp 3′-UTR found in these species was different from other RyRs that reported, as it is the longest 3′-UTR found to date. The deduced 5,133 amino acid sequence of *Gm*RyR showed higher amino acid identity (91–93%) with reported Lepidopteran insect RyRs compared other insect RyRs (78–79%). Furthermore, Lepidopteran insect RyRs share only 44–47% amino acid identities with mammalian isoforms. Therefore, the selectivity of diamide insecticides toward insect RyRs was suggested to be due to differences in RyR types between insects and mammals (Wang et al. 2012b). The spectrum of activity for diamides is wide, with flubendiamide targeting mostly Lepidoptera, while chlirantraniliprole and cyantraniliprole are effective in controlling Homoptera, Coleoptera, Diptera, and Thysanoptera species in addition to Lepidoptera (Teixeira and Andaloro 2013).

A multiple alignment of the C-terminal amino acid sequence from *Gm*RyR with other reported RyRs, including BmRyR, PxRyR, OfRyR, HvRyR, DmRyR, and OcRyR1 (*Or. cuniculus* RyR), showed that the TM regions had high identities among the insect RyR isoforms aligned. The fragment motif, GVRAGGGIGD, sitting at residues 4,985–4,994 between TM5 and TM6 is known to form part of the pore-forming segments of the RyR Ca^2+^ release channel (Zhao et al. 1999). This motif from *Gm*RyR shared complete identity with other all RyR isoforms. Moreover, the residue that enables Ca^2+^ sensitivity in the C-terminal domain of rabbit RyR1 (E4032) was also detected in *Gm*RyR (E4170). Residues corresponding to I4897, R4913, and D4917 in OcRyR1, which were shown to play an important role in the activity and conductance of the RyR Ca^2+^ release channel, were also conserved in *Gm*RyR (I4992, R5008, and D5012) (Zorzato et al. 1990).

Three binding regions have been predicted to be critical to diamide insecticide sensitivity. One region lies near the N-terminus (residues 183–290 from *Bombyx* *mori*) and two are located in the C-terminal TM domain (residues 4,610–4,655 from Drosophila and residue 4,946 from *P. xylostella*) (Kato et al. 2009, Troczka et al. 2012, Tao et al. 2013). Flubendiamide was identified to incorporate into the TM domain (amino acids 4,111–5,084) of *Bm*RyR (Kato et al. 2009). Residues 188–295 of *Gm*RyR share 95% identity and are equivalent with the 183–290 region of BmRyR; they both share 47–50% identity with mammalian RyRs. However, there were three sites in two regions with significant differences among species for the equivalent residues 188–295 of *Gm*RyR (Table 3). AaRyR and DmRyR have a different residue in region I, three nematode species have three different residues in region II, and HsRyR1, MmRyR1, OcRyR1, and MgRyRα were different from other vertebrate RyRs. Table 3 indicated that region I residues were EV in Lepidoptera insects except for the peach fruit moth CsRyR, where they were EI. Region I residues were highly variable in other species. Region II residues contain eight amino acids, which have significant differences in all insects, except Noctuidae. Moreover, seven residues (N4922, N4924, N4935, L4950, L4981, N5013, and T5064) are unique to RyR isoforms from Lepidopteran insects (Wang et al. 2012a). Wang et al (2012a) and Cui et al. (2013) speculated that these residues might contribute to the differences in Ca^2+^ release channel properties between Lepidopteran and non-Lepidopteran insects RyRs (Wang et al. 2012a, Cui et al. 2013). However, we have different thoughts from Wang and Cui due to the insecticidal spectrum of diamides. Four special residues (D4452, P4504, V4647, and I4758) in residues 4,146–5,133 of *Gm*RyR corresponding to residues 4,111–5,084 of BmRyR are shown in Table 2. Each residue is different among insects RyRs, nematode RyRs, and invertebrate RyRs. We think that these four residues might be involved in Ca^2+^ release from channels among Lepidoptera, non-Lepidoptera, nematode, and invertebrate RyRs. Isolation of microsomal membranes from insect muscles suggest that flubendiamide and chlorantraniliprole target a site localized in the pore of the insect RyR complex distinct from the ryanodine-binding site, suggesting that diamides can bind to several sites in RyR at the same time.

**Table 3.** Differences of RyRs in the N-terminal and TM domains

		Animo acid position^#^								
		114	129	151	188–189	240–247	4,452	4,504	4,647	4,758
Lepidoptera	*Gm*RyR	L	Q	L	EV	TWSTEGGQ	D	P	V	I
	CsRyR	L	Q	L	EI	TWSKEGGQ	D	P	V	I
	SeRyR	L	Q	L	EV	TWTKDGGQ	D	P	V	I
	HvRyR	L	Q	L	EV	TWTKDGGQ	D	P	V	I
	HaRyR	L	Q	L	EV	TWTKDGGQ	D	P	V	I
	BmRyR	L	Q	L	EV	TWTRDGGQ	D	P	V	I
	OfRyR	L	Q	L	EV	AWAKETGQ	D	P	V	I
	CmRyR	L	Q	L	EV	TWTKDGGL	D	P	V	I
	PxRyR	L	Q	L	EV	SWSNEGQH	D	P	V	I
	PrRyR	L	Q	L	EV	TWNKDGGL	D	P	V	I
Coleoptera	LdRyR	Q	N	V	EI	TWDMEPGH	E	V	I	M
Homoptera	NlRyR	Q	N	V	DL	TWSEAPGQ	E	V	I	M
	SfRyR	Q	N	V	DL	TWSEAPGQ	E	V	I	M
	LsRyR	Q	N	V	DL	TWSEAPGQ	E	V	I	M
	BtRyR	Q	N	V	DL	NWTETTGQ	E	V	I	M
Diptera	AaRyR	N	N	V	DL	TWGQEPGQ	V	E	I	M
	DmRyR	H	N	V	EQ	TWGREAGQ	V	E	I	M
Nematode	TtRyR	V	T	I	MT	WSDHSQQN	S	V	V	L
	CeRyR	V	N	I	YM	NWSEHPQH	S	A	S	L
	SrRyR	V	S	I	DQ	WSENQIHN	K	I	I	L
Vertebrate	HsRyR1	A	T	M	GE	ADS-DDQR	R	T	G	C
	MmRyR1	A	T	M	GE	SDS-DDQR	R	T	G	C
	OcRyR1	A	T	M	GE	ADS-DDQR	R	T	G	C
	MgRyRα	S	T	L	GE	PEQGDERS	K	A	T	C
	RcRyRα	C	T	I	GD	TDQGEEQR	K	T	N	C
	HsRyR2	S	T	I	GS	GEHGEEQR	K	A	K	C
	MmRyR2	S	T	I	SS	GEHGEEQR	K	A	K	C
	OcRyR2	S	T	I	GS	GEHGEEQR	K	A	K	C
	HsRyR3	S	T	I	GN	TDQNDSQH	K	T	K	C
	MmRyR3	S	T	I	GS	TDQNDSQH	K	T	K	C
	OcRyR3	S	T	I	GN	TDQNDSQH	K	T	K	C
	MgRyRβ	S	T	I	GS	TDQGEEQR	K	T	K	C
	RcRyRβ	S	T	I	GN	TDQGEEQR	K	T	A	C

^#^The number of amino acid M in the initial ORF from GmRyR represents 1.

The putative adenine ring binding domain Y[GAST][VG] [KTQSN] was found at two locations (residues 1,145–1,148 [YSGS] and 5,098–5,101 [YTGQ]) in *Gm*RyR. Two possible nucleotide-binding sites, identified on the basis of the consensus GXGXXG motif (Wierenga and Hol 1983), were located at positions 3,999–4,004 (GVGLEG) and 4,717–4,722 (GSGESG) in the *Gm*RyR sequence. A potential calmodulin-binding site in *Gm*RyR was recognized in residues 3,745–3,774, corresponding to residues 3,614–3,643 in OcRyR1 (Xiong et al. 1998). In addition, the binding sites of adenine nucleotide have no particularities compared with other Lepidopteran RyRs, such as *O. furnacalis*, *C. medinalis*, and *P. xylostella* (Wang et al. 2012a, Sun et al. 2012, Cui et al. 2013).

The presence of each putative alternative splice variant and expression of *GmRyR* mRNA in five discrete mRNA pools (larva, female pupa, male pupa, female adult, and male adult) were determined (Fig. 4). The alternative splice segments were different from *CmRyR*, *OfRyR,* BdRyR, and *NlRyR* (Wang et al. 2012, 2013; Cui et al. 2013; Yuan et al. 2014). Five alternative splice segments were located between amino acid residues 89–93 (a), 1,141–1,173 (b/c), 1,487–1,507 (d), 2,949–2,954 (e), and 3,699–3,706 (f). Interestingly, only the mutually exclusive exons (b/c) were also found in *CmRyR*, *OfRyR,* BdRyR, and *NlRyR* and were developmentally regulated (Wang et al. 2012a, 2014; Cui et al. 2013; Yuan et al. 2014). Furthermore, alternative splicing of residues (Ala^3481^-Gln^3485^) in RyR1 was thought to be developmentally regulated (Kimura et al. 2009). The BLAST results showed that residues 89–93 were present in CsRyR, PxRyR, HaRyR, HvRyR, and OfRyR. The analysis of alternatively spliced regions revealed that region b/c and d were located in the central part of the predicted SPRY domain and region e was located between the third and fourth RyR domains. The alternatively spliced region f was only in pupae and adults from *Gm*RyR. Splice variants of RyR2 have been recognized for over a decade but their functions are as undefined to date. Moreover, Knight and Flexner (2007) confirmed that males were more sensitive to chlorantraniliprole-related disruption of mating than females. Thus, further studies are needed to elucidate the characterization and functions of each *Gm*RyR splice variant.

There are some differences in RyR expression between invertebrates and vertebrates. RNA from eggs of *G. molesta* was not extracted because the eggs were not successfully collected. Real-time quantitative PCR showed that the expression level of *Gm*RyR mRNA was quite different in larvae, pupae, and adults, particularly in pupae and adults of different sexes. The results showed the lowest expression level in larvae and the highest in male pupae. Wang et al. (2014) reported that *NlRyR* mRNA expression levels in macropterous female adults were significantly higher than that in brachypterous female adults as well as macropterous and brachypterous male adults. Guo et al. (2012) reported that the expression abundance of the RyR in the second-instar larvae and adults were considerably higher than those of the prepupae and pupae in *P. xylostella*. Wang et al. (2012b) showed that *PxRyR* expression level in larvae was higher than in pupae. The expression levels of *OfRyR* were gradually upregulated with increasing age (Cui et al. 2013), and different expression levels of RyR were shown in *BdRyR* (Yuan et al. 2014). In summary, although we cloned and characterized the expression levels of *Gm*RyR, its properties and functions remain to be studied.
